# Cardio-renal protective effect of the xanthine oxidase inhibitor febuxostat in the 5/6 nephrectomy model with hyperuricemia

**DOI:** 10.1038/s41598-020-65706-6

**Published:** 2020-06-09

**Authors:** Hiroki Omizo, Yoshifuru Tamura, Chikayuki Morimoto, Masaki Ueno, Yuto Hayama, Emiko Kuribayashi-Okuma, Shunya Uchida, Shigeru Shibata

**Affiliations:** 1 Division of Nephrology, Department of Internal Medicine, Teikyo University School of Medicine, 2-11-1 Kaga, Itabashi-ku, Tokyo, 173-8605 Japan; 2 Department of Health Care, Teikyo Heisei University, 2-51-4 Higashi-Ikebukuro, Toshima-ku, Tokyo, 170-8445 Japan

**Keywords:** Nephrology, Kidney diseases, Translational research

## Abstract

Although hyperuricemia has been shown to be associated with the progression of cardiovascular disorder and chronic kidney disease (CKD), there is conflicting evidence as to whether xanthine oxidase (XO) inhibitors confer organ protection besides lowering serum urate levels. In this study, we addressed the cardio-renal effects of XO inhibition in rodent CKD model with hyperuricemia. Sprague-Dawley rats underwent 5/6 nephrectomy and received a uricase inhibitor oxonic acid for 8 weeks (RK + HUA rats). In some rats, a XO inhibitor febuxostat was administered orally. Compared with control group, RK + HUA group showed a significant increase in albuminuria and renal injury. Febuxostat reduced serum uric acid as well as urinary albumin levels. Histological and immunohistochemical analysis of the kidney revealed that febuxostat alleviated glomerular, tubulointerstitial, and arteriolar injury in RK + HUA rats. Moreover, in the heart, RK + HUA showed individual myofiber hypertrophy and cardiac fibrosis, which was significantly attenuated by febuxostat. We found that renal injury and the indices of cardiac changes were well correlated, confirming the cardio-renal interaction in this model. Finally, NF-E2-related factor 2 (Nrf2) and the downstream target heme oxygenase-1 (HO-1) protein levels were increased both in the heart and in the kidney in RK + HUA rats, and these changes were alleviated by febuxostat, suggesting that tissue oxidative stress burden was attenuated by the treatment. These data demonstrate that febuxostat protects against cardiac and renal injury in RK + HUA rats, and underscore the pathological importance of XO in the cardio-renal interaction.

## Introduction

Chronic kidney disease (CKD) has increasingly been recognized as an important contributor not only of end-stage kidney disease but also of cardiovascular disease (CVD). Decreased glomerular filtration rate (GFR) and albuminuria increase the risk of CVD independently of other atherosclerosis risk factors^1^, and CVD is the leading cause of deaths at all stages of CKD^2^. Although the frequent association of CVD with CKD suggests the pathogenic link between these conditions, the underlying mechanisms remain unclear. Besides several common risk factors of CKD and CVD such as hypertension, ischemia, and impaired glucose tolerance, several lines of evidence indicate that the disturbed uric acid (UA) metabolism may mediate cardio-renal syndrome^3^. In CKD, the reduced excretion of UA from the kidney results in the elevation of serum UA levels, and we have previously demonstrated that hyperuricemia, in turn, contributes to the progression of kidney injury^4,5^. Importantly, hyperuricemia has also been reported to be associated with increased risk for incident coronary heart disease and heart failure^6,7^, supporting that UA is one of the key factors of the cardio-renal interaction.

Given the possible role of hyperuricemia in the progression of CKD and CVD, a potential benefit for the xanthine oxidase (XO) inhibition has been studied^8–10^; however, clinical data to date are still controversial. A recent cohort study comparing gout patients on XO inhibitors (XOIs) with non-treated subjects who have hyperuricemia showed that XOIs had no effect on cardiovascular risk^11^. In another study, the administration of a XOI, febuxostat, did not show significant renoprotective effect in hyperuricemic stage 3 CKD patients^12^. In contrast, in a very recent report, febuxostat was shown to reduce the primary composite endpoint of cerebral, cardiovascular, and renal events and all deaths as compared with non-febuxostat group in patients with 65 years or older with hyperuricemia^13^. In the Cardiovascular Safety for Febuxostat and Allopurinol in Patients with Gout and Cardiovascular Morbidities (CARES) trial, febuxostat was noninferior with respect to adverse cardiovascular events^14^. However, cardiovascular mortality was higher with febuxostat than with allopurinol in patients with gout and cardiovascular disease. These inconsistent results may be due to the differences in study design, baseline characteristics, and the rate of GFR decline^15^. Currently, it is still inconclusive whether XOIs can confer organ protection besides reducing circulating UA levels.

Previously, we demonstrated that the disturbed UA metabolism is associated with albuminuria and glomerular podocyte injury in experimental hyperuricemic rats^5^. However, it was unclear whether the use of XOIs was able to confer cardio-renal protection. In this study, we tested whether XO inhibition ameliorates cardiovascular and renal dysfunction in a model of CKD with hyperuricemia.

## Materials and methods

### Animal experiments

Animal procedures were approved by the Teikyo University Ethics Committee for Animal Experiments (Animal Ethics Committee, No. 18-030) and were conducted in accordance with the guidelines of the Institute Animal Care and Use Committee of the Teikyo University. Male Sprague Dawley rats at 6 weeks of age were obtained from Sankyo Labo Service (Tokyo, Japan). After baseline blood pressure (BP) measurement, rats were randomly assigned to the remnant kidney (RK) group or the sham-operated control group. RK model was created as described previously^16^. In brief, rats received the surgical resection of the upper and lower one-thirds of the left kidney. The resected portion of the left kidney was weighed to validate the procedure. One week later, the rats received right uninephrectomy. Rats were divided into three subgroups: 1) Control group (n = 6), 2) RK with oxonic acid group (n = 5; RK + HUA), 3) RK with oxonic acid and Febuxostat group (n = 6; RK + HUA + Feb). Control rats received standard diet (CRF1, Oriental Yeast, Tokyo, Japan) and tap water. RK rats received oxonic acid (Sigma, St. Louis, MO), a uricase inhibitor (2 g/100 g chow; the dose was decided according to the previous studies)^17^. Febuxostat was dissolved in drinking at a concentration of 0.03 mg/L. At this concentration, the dose of febuxostat was calculated as ~3 mg/kg/day^18^. Systolic BP was measured by the tail-cuff method (Model MK-2000; Muromachi Kikai, Tokyo, Japan). Urine was collected for 24 hours using individual metabolic cages. At 8 weeks, animals were euthanized under anesthesia using inhaled isoflurane for sample collection. Blood samples were obtained by Inferior vena cava. Kidney and heart were removed and were snap-frozen until use.

### Laboratory studies

Serum UA concentrations were determined using high-performance liquid chromatograph equipped with a UV spectrophotometric detector (Prominence; Shimadzu, Kyoto, Japan). UA standard (final concentration of 5 mg/dL) was dissolved in 2 mmol/L ammonium hydroxide solution. Serum samples were centrifuged and filtered through a Millipore filter (0.22 μm pore size; Darmstadt, Germany). Samples were injected onto a Wakopak Wakosil GP-N6 column (which enables direct analysis of a specimen without deproteinization; 15 × 4.6 mm ID) with mobile phase of 98% (v/v) 0.2 mol/L sodium phosphate buffer, pH6.0, and 2% (v/v) acetonitrile at a flow rate of 0.5 mL/min. Under these conditions typical retention time for uric acid (detected at 284 nm) was 3.68 min. Urinary albumin, urinary creatinine, and serum creatinine were measured by ELISA (SRL, Tokyo, Japan).

### Histomorphological analysis and immunohistochemistry

Methyl Carnoy’s solution-fixed, paraffin-embedded sections (1 μm) were stained with the periodic acid-Schiff (PAS) reagent for light microscopy. On coronal sections of the kidney, at least 20 glomeruli were examined to evaluate glomerular hypertrophy as described previously^19^.

Immunohistochemistry was performed as described previously^19^. Primary antibody included antibodies against desmin (DAKO, Denmark), type III collagen (Southern Biotech, USA), and α-SMA (Sigma, USA). To assess the desmin-positive area, the digital images at ×400 magnification were analyzed using Aperio ImageScope. The percent positive area was determined as the diaminobenzidine-positive pixel per total pixel in the glomerulus. Likewise, positive area for type III collagen in interstitium was determined as percent positive area with 15 fields (each field/0.4 mm^2^). Quantitative analysis of afferent arterioles was performed as described previously^20^. For afferent arterioles, vessels with internal elastic lamina adjacent to glomeruli were selected. Areas positive for α-SMA in the cross section of these vessels were quantitated using Aperio ImageScope (v12.4, Leica, Vista, CA, USA). Kidney sections were observed by two investigators in a blinded manner.

For the histological evaluation of the heart, short-axis slices of the heart at the level of papillary muscle were washed with saline, immersed in 10% Mildform, fixed for one day, and embedded in paraffin. Hematoxylin and eosin (HE)-stained heart sections were used to quantify myocardial thickness by measuring the distance from the inner to the outer myocardial edges at the mid-papillary level. Mean left ventricular wall thickness was calculated from seven measurements of wall thickness taken at 0, 30, 60, 90, 120, 150, and 180 degrees along the hemicircle of the short axis of the left ventricular wall, and the average was used to calculate mean ventricular thickness. Myofibril cross-sectional areas were evaluated using wheat germ agglutinin (WGA)-stained sections^21^. We quantified cross-sectional area of 25 cells per field at 4 fields. Additionally, heart sections were stained with sirius-red stain to distinguish areas of connective tissue as previously described^22^. Positive areas were quantified using Aperio ImageScope (eight fields were randomly selected on each section). The value was expressed as the ratio of sirius red-stained fibrosis area to total area.

### Western blotting

Kidney cortex and heart were homogenized in cell lysis buffer (Cell Signaling, Danvers, MA) and Western blotting was performed as described previously^19^. Briefly, samples were subjected to SDS-PAGE and transferred onto a nitrocellulose membrane. After overnight incubation with primary antibody at 4 °C, membrane was incubated with secondary antibody linked with horseradish peroxidase for 1 h at RT. Signal was detected by Immun Star HRP (Bio-Rad, Hercules, CA). The density of each band was determined using Multi Gauge software (Fuji film, Tokyo, Japan) and expressed as a value relative to the density of the corresponding band of the GAPDH. As primary antibodies, Nrf2 antibody (abcam, Cambridge, UK) and heme oxygenase-1 (HO-1) antibody (Enzo Life Sciences, NY, USA) were used. Full-length blots are shown in Supplemental Fig. 1.

### Statistical analysis

All values are expressed as mean ± SD. Comparison of continuous variables among three groups was performed by ANOVA, followed by Tukey’s HSD post hoc test. Linear regression analysis was performed by Pearson correlation. All statistical analyses were conducted using Graph Pad Prism 7 (GraphPad Software, USA). A value of P < 0.05 was considered statistically significant.

## Results

### Biological data

**Figure 1:**
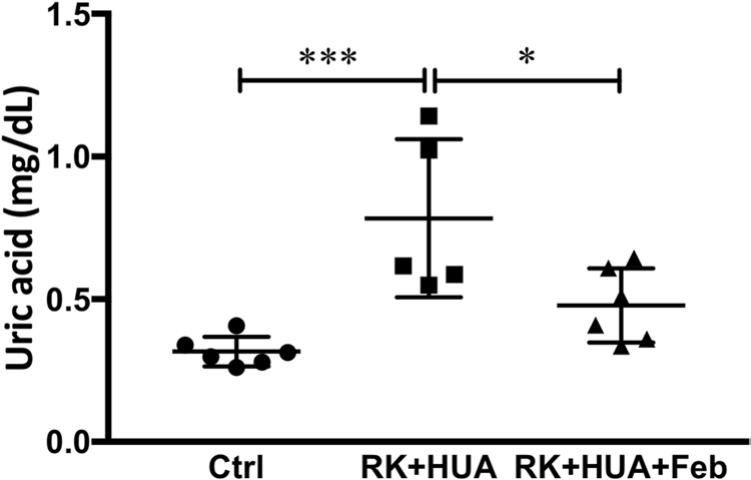
Serum uric acid levels. Serum uric acid (UA) levels were measured by high-performance liquid chromatography at 8 weeks in control rats (Ctrl), 5/6 nephrectomized, hyperuricemic rats (RK + HUA), and RK + HUA rats receiving febuxostat (RK + HUA + Feb). Data are expressed as mean ± SD; *n* = 5 or 6 per group; *P < 0.05; ***P < 0.001.

Male Sprague Dawley rats were randomly assigned to one of three subgroups: 1) control group (n = 6), 2) remnant kidney (RK) with oxonic acid group (n = 5; RK + HUA), 3) RK with oxonic acid and febuxostat group (n = 6; RK + HUA + Feb) (see Methods). Biological data in each group at 8 weeks are shown in Table 1. Compared with control, RK + HUA and RK + HUA + Feb rats had a significant decrease in creatine clearance. Serum UA levels were significantly elevated in the RK + HUA group compared to the control group, and these changes were significantly attenuated in RK + HUA + Feb group (Fig. 1). Systolic BP was significantly increased in RK + HUA group compared with the control group. BP was partially reduced in RK + HUA + Feb, although the difference did not reach statistical significance (Table 1).

**Table 1:** Blood pressure, Aldosterone, renal function, body and heart weight data, Urinary 8-OHdG at 8 weeks.

	Ctrl (N = 6)	RK + HUA (N = 5)	RK + HUA + Feb (N = 6)
Systolic blood pressure (mmHg)	121 ± 5	141 ± 13**	130 ± 10
Creatinine clearance (ml/min)	3.12 ± 0.54	1.37 ± 0.24***	1.47 ± 0.33***
Body weight (g)	482 ± 38	368 ± 24***	367 ± 19***
Heart weight/Body weight (mg/g)	2.38 ± 0.13	2.95 ± 0.09**	2.91 ± 0.35**
Serum aldosterone (pg/ml)	369 ± 73.1	391 ± 201	309 ± 94.9

Ctrl, control group; RK, remnant kidney; HUA, oxonic acid-treated; Feb, rats treated with febxostat; RK + HUA, RK rats treated with oxonic acid; RK + HUA + Feb, RK + HUA + Feb; RK + HUA rats treated with febxostat.

Data are expressed as mean ± SD. ***P 0.001 vs. Ctrl, **P 0.001 vs. Ctrl.

### Effects of febuxostat on renal injury

**Figure 2:**
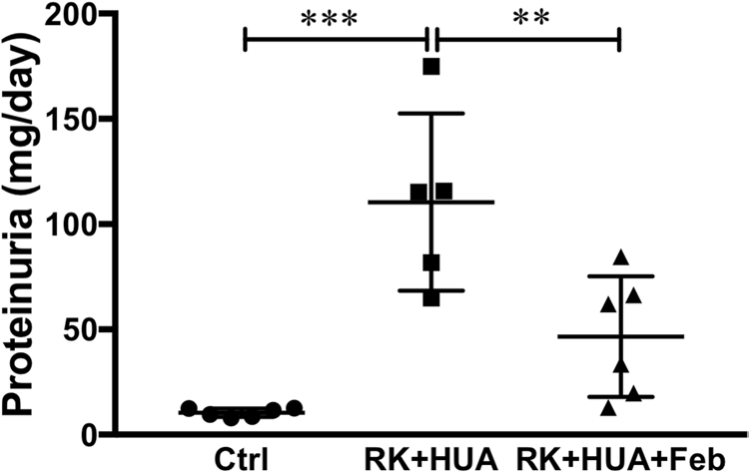
Urinary protein levels. Urinary protein excretion measured at 8 weeks in Ctrl, RK + HUA, and RK + HUA + Feb groups. Data are expressed as mean ± SD; *n* = 5 or 6 per group; **P < 0.01; ***P < 0.001.

**Figure 3:**
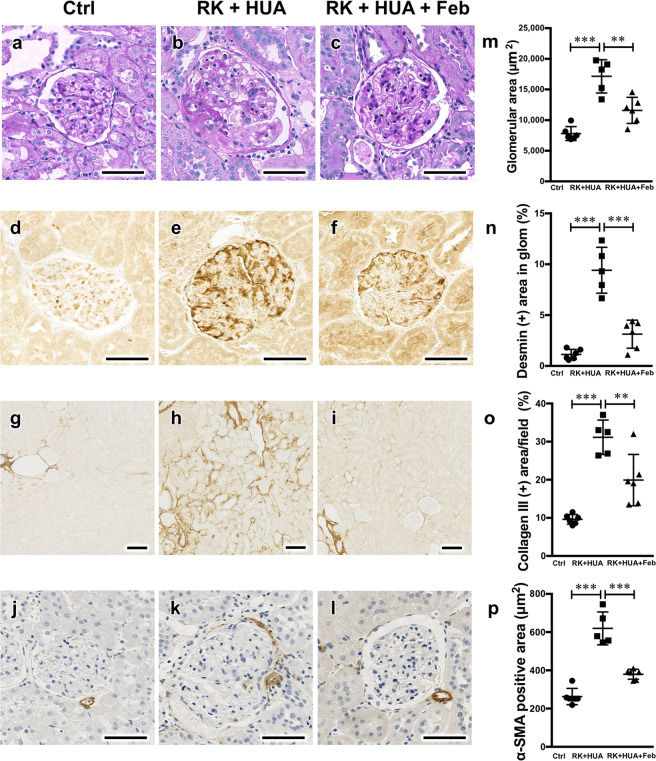
Renoprotective effects of febuxostat in RK + HUA model. (**a–c**) Representative Periodic acid-Schiff (PAS)-stained kidney sections from Ctrl, RK + HUA, and RK + HUA + Feb rats. (**d–f**) Representative micrographs of immunostaining for desmin, a marker for podocyte injury. (**g–i**) Representative micrographs of immunostaining for collagen III. (**j–l**) Kidney sections were stained for α-smooth muscle actin (α-SMA) to evaluate the thickening of afferent arterioles. (**m**) Dot plots for histological analysis of glomerular hypertrophy. (**n**) Dot plots for quantitative evaluation of desmin staining in the glomeruli. (**o**) Dot plots for quantitative evaluation of collagen III staining in the kidney. (**p**) Dot plots for quantitative evaluation of α-SMA-positive areas in the renal arterioles. Data are expressed as mean ± SD; *n* = 5 or 6 per group; **P < 0.01; ***P < 0.001.

We evaluated renal injury in our model. Compared with control group, RK + HUA rats showed a significant increase in proteinuria (Fig. 2). The administration of febuxostat reduced proteinuria in this model by 57.9% (P = 0.004). In the PAS-stained kidney section, RK + HUA group showed glomerular enlargement, which was significantly attenuated in RK + HUA + Feb rats (Fig. 3a–c,m). Glomerular epithelial cells, or also called podocytes, are the specialized cells that cover the external surface of the glomerular basement membrane and serve as the filtration barrier for the plasma protein. Because we found significant changes in proteinuria in our model, we also addressed podocyte injury. Compared with the control group, RK + HUA showed marked upregulation of desmin, a marker of podocyte injury^23^ (Fig. 3d–f,n). However, these changes were significantly alleviated in RK + HUA + Feb group. We also evaluated interstitial fibrosis and renal arteriole injury by collagen III staining and by α-smooth muscle actin (α-SMA), respectively. Compared with the control group, RK + HUA group showed significant interstitial fibrosis, which was attenuated in RK + HUA + Feb group (Fig. 3g–i,o). Thickening of the vascular smooth muscle cells in renal arterioles in RK + HUA group were also significantly reduced by febuxostat (Fig. 3j–l,p). Thus, febuxostat was capable of attenuating glomerular injury, renal fibrosis and arteriole damages in the hyperuricemic CKD model.

### Effects of febuxostat on cardiac injury and its association with renal damage

**Figure 4:**
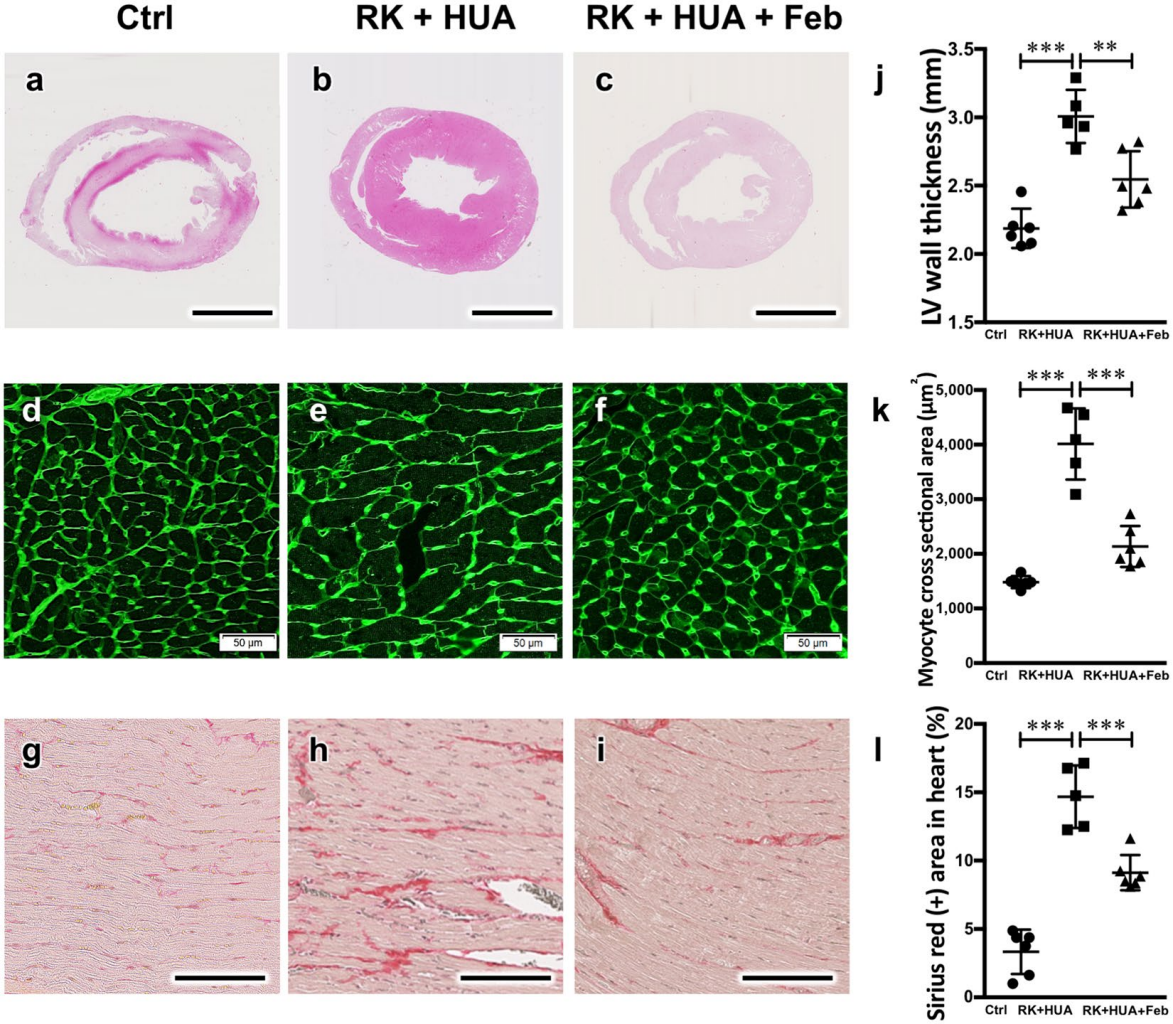
Attenuation of cardiac hypertrophy and fibrosis by febuxostat in RK + HUA model. (**a–c**) Representative HE-stained cardiac sections from Ctrl, RK + HUA, and RK + HUA + Feb rats. (**d–f**) Wheat germ agglutinin (WGA) staining for heart cross-sections. (**g–i**) Sirius red staining in the heart to evaluate cardiac fibrosis. (**j**) Quantitative evaluation of left ventricular wall thickness. (**k**) Quantitative analysis of the cardiomyocyte cross-sectional area (see Methods). (**l**) Dot plots for quantitative evaluation of Sirius red-positive areas in the heart. Data are expressed as mean ± SD; *n* = 5 or 6 per group; **P < 0.01; ***P < 0.001.

Next, we performed structural analysis of the cardiac tissues in our model. In HE staining, RK + HUA showed significant thickening of the left ventricular wall, which was partially but significantly reduced by febuxostat administration (Fig. 4a–c,j). Consistent with these data, WGA staining revealed that the individual myofibril diameter in the cross-sectional areas was increased in RK + HUA rats, and that febuxostat administration attenuated the changes (Fig. 4d–f,k). Furthermore, cardiac fibrosis, as assessed by Sirius red staining, was alleviated by febuxostat (Fig. 4g–i,l).

**Figure 5:**
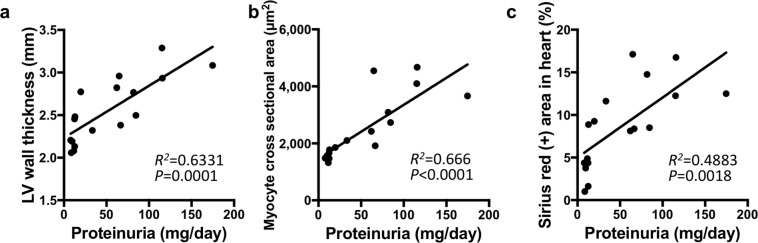
Correlations of urinary albumin levels with structural changes of the heart. Correlations of albuminuria with left ventricular (LV) wall thickness (**a**), myocyte cross sectional area as evaluated by WGA staining (**b**), and Sirius red-positive area (**c**) are shown. The correlation coefficient and P value are shown in each panel.

To determine the cardio-renal interaction in our model, we then addressed the association of cardiac injury markers with renal damage across control, RK + HUA, and RK + HUA + Feb groups. Correlation analysis demonstrated that proteinuria, a surrogate marker for renal injury^24^, was highly correlated with LV wall thickness (R^2^ = 0.633; P < 0.001) and with myofibril hypertrophy (R^2^ = 0.666; P < 0.001) (Fig. 5a,b). In addition, it also moderately correlated with fibrotic areas in cardiac tissue (R^2^ = 0.488; P = 0.002) (Fig. 5c). These data demonstrate that XOIs can protect cardiac damage in our model, and indicate the involvement of XO in cardio-renal interaction.

### Possible role of oxidative stress in cardiac and renal damage in hyperuricemic CKD rats

**Figure 6:**
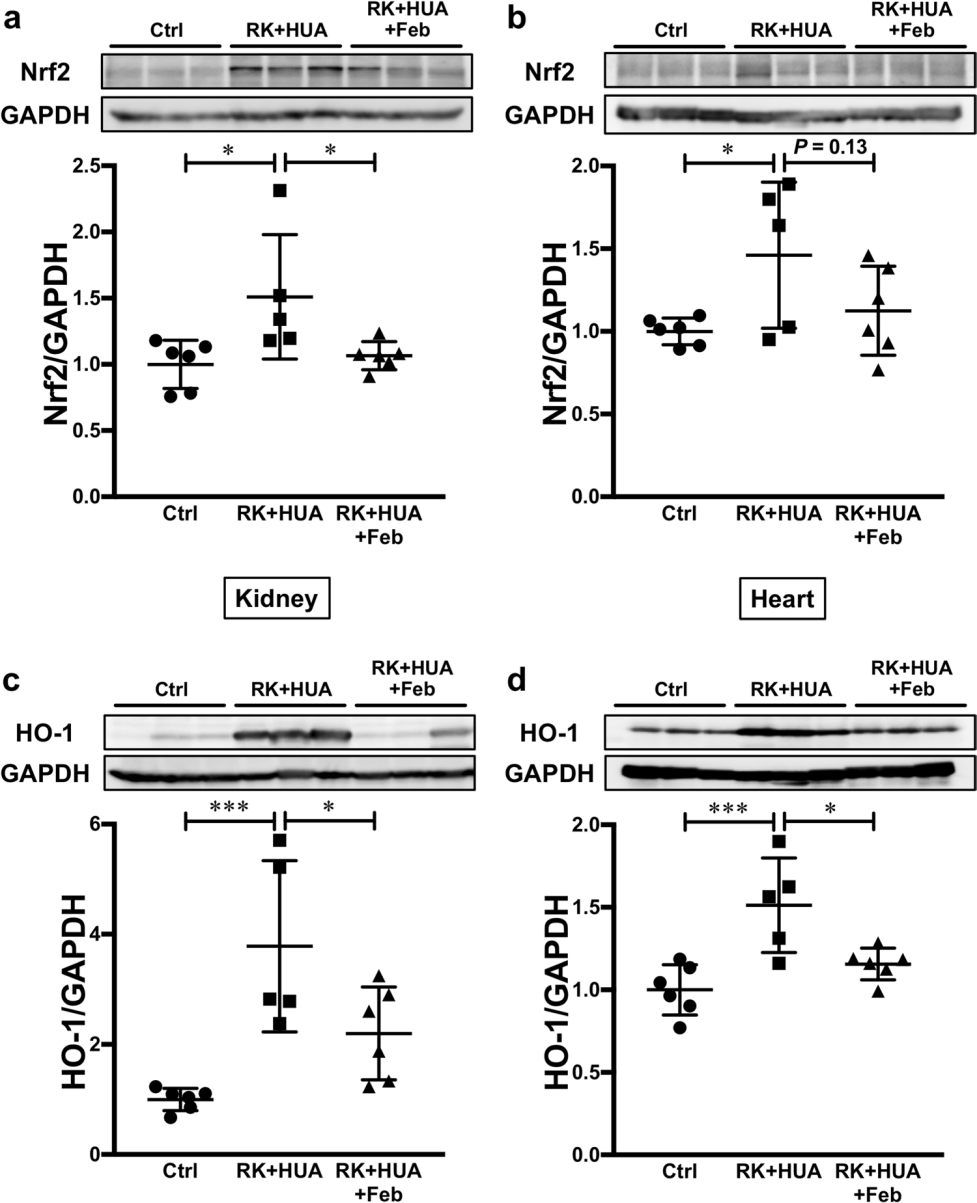
Evaluation of Nrf2 and HO-1 abundance in the kidney and in the heart. (**a**,**b**) The levels of Nrf2 in the kidney (**a**) and heart (**b**) were evaluated by Western blotting in Ctrl, RK + HUA, and RK + HUA + Feb groups. GAPDH was used as loading control. Dot plots show the results of quantitation. (**c**,**d**) HO-1 levels in the kidney (**c**) and heart (**d**) in Ctrl, RK + HUA, and RK + HUA + Feb groups were evaluated by Western blotting. Dot plots show the results of quantitation. Data are expressed as mean ± SD; *n* = 5 or 6 per group; *P < 0.05; ***P < 0.001.

Finally, we evaluated possible mechanisms for the protective effects of febuxostat in our model. CKD has been shown to be associated with oxidative stress accumulation^25^. Several lines of evidence also indicated that increased UA levels can also result in intracellular oxidative stress^5,26,27^. In addition, XOIs not only reduce UA production during purine metabolism, they also decrease oxidant stress formation such as H_2_O_2_ and O_2_^−^. The Kelch-like ECH-associated protein 1 (Keap1)-NF-E2-related factor 2 (Nrf2) system serves as the physiological sensor of cellular oxidative stress^28,29^. In response to increased reactive oxygen species, covalent modification of several key cysteine residues in the ubiquitin ligase component Keap1 impairs the binding to the substrate Nrf2, resulting in its accumulation. Increased Nrf2, in turn, induces antioxidant proteins such as heme oxygenase 1 (HO-1)^28,29^. To determine oxidative stress burden in our model, we evaluated Nrf2 levels in the kidney and in the heart by Western blotting. We found that Nrf2 levels were significantly increased in the kidney as well as in the heart in RK + HUA group (Fig. 6a,b). However, the elevation in Nrf2 levels was significantly attenuated in the kidney, and non-significantly attenuated in the heart in RK + HUA + Feb group. To confirm the altered Nrf2 activity in our model, we also evaluated the levels of HO-1, the key downstream target of Keap1-Nrf2 system. Consistently, we found that HO-1 levels were significantly increased both in the kidney and in the heart of RK + HUA rats compared with control (a 3.3-fold increase and a 1.6-fold increase, respectively; Fig. 6c,d). However, these changes were significantly attenuated in the kidney and in the heart in RK + HUA + Feb rats (a 50.3% reduction and 32.4% reduction, respectively, compared with RK + HUA group). These data indicate that the increased oxidative stress in the kidney and heart in hyperuricemic CKD rats was effectively reduced by febuxostat, explaining the mechanisms for the protective effects.

## Discussion

In this study, we demonstrated that febuxostat attenuates renal and cardiac injury in hyperuricemic CKD model rats. We also showed the significant association between cardiac and renal injury in our model, confirming the pathogenic link between the two organs. Previously, several studies examined the protective effects of febuxostat in experimental models of chronic kidney injury. Similar to our study, Sanchez-Lozada *et al*. reported that proteinuria, tubulointerstitial injury and renal arteriolopathy in hyperuricemic remnant kidney model were attenuated by febuxostat^30^. In another model, the effects of febuxostat were tested in rats receiving oxonic acid and tacrolimus, a calcineurin inhibitor^31^. In this study, renal arteriolopathy, inflammation, and interstitial fibrosis were ameliorated by febuxostat. The renoprotective effect of febuxostat was also reported in streptozotocin-induced diabetic rat model^32^ and in KK-Ay obese diabetic mice^33^. However, in these studies, changes in cardiac morphology and the effects of febuxostat on cardio-renal interaction were not determined. The data described herein showed that the cardiac hypertrophy and fibrosis are associated with the degree of proteinuria, and that febuxostat effectively attenuates both cardiac and renal injury, suggesting the key role of XO in cardio-renal syndrome.

Another interesting observation is an altered activity of Keap1-Nrf2 system in our model. In a basal condition, the abundance of the transcription factor Nrf2 is suppressed through the proteasomal degradation that is controlled by the oxidative stress sensor Keap1^28,29^. However, increased oxidative stress abrogates substrate-binding ability of Keap1, increasing Nrf2 levels and downstream targets; thus, Nrf2 accumulation indicates the increased cellular oxidative stress in target organs. In this study, we observed the increase in Nrf2 as well as the downstream target HO-1 in the kidney and in the heart in hyperuricemic CKD rats at 8 weeks. However, previous studies using rat remnant kidney model showed that Nrf2 is reduced at a later stage (12 weeks)^34^. Given these results, it may be that the anti-oxidant mechanism is upregulated in response to oxidative stress at an early stage of CKD, however the response to oxidant stress is compromised at an advanced stage possibly due to the accumulation of uremic toxins, facilitating oxidative stress accumulation and tissue damage. Currently, several clinical trials that evaluate the protective effects of Nrf2 activators are ongoing^35^. These studies will clarify the role of the Keap1/Nrf2 system in CKD progression.

In this study, febuxostat induced a non-significant decrease in BP in hyperuricemic CKD rats. XO has been shown to be widely distributed across the body, including liver, kidney, heart, and capillary endothelial cells^36^. It has also been shown that plasma XO activity is increased in hypertensive subjects, which is associated with cardiac hypertrophy^37^. Consistently, XOIs have been shown to reduce BP in hypertensive animal models^17,38^. In addition, the BP-lowering effects of XOI have also been reported in humans^39^. Our data are in line with these data, and the protective effects of febuxostat observed in our study may in part be explained by the reduction in BP. However, the fact that the BP difference between RK + HUA and RK + HUA + Feb rats did not reach statistical significance also points to the additional contribution of BP-independent factors.

Accumulating data indicate that tissue damage associated with the disturbed UA metabolism is mediated by both urate crystal-dependent and -independent mechanisms^40^. The increase in extracellular UA levels above the solubility facilitates the formation of monosodium urate crystals, which triggers tissue inflammation through NALP3 inflammasome and other pathways^41^. Besides these mechanisms, several lines of evidence indicate that intracellular urate triggers a number of pathological mechanisms such as the activation of renin-angiotensin system, NADPH oxidase, and endothelin-1^26,40,41^. In addition, XO generates H_2_O_2_ and O_2_^−^ in the process of purine metabolism, which is also implicated in tissue damage^42^. In accordance with previous observations^5,17^, we did not observe any crystal formation in the kidney (data not shown). This is explained by the fact that serum UA levels in the hyperuricemic rats were well below the maximal solubility in the extracellular milieu, although the levels were increased by approximately two fold compared with control group. In our study, contribution of oxidative stress is indicated by the activation of Nrf2 both in the kidney and in the heart; however, future studies are required to determine to what extent the intracellular UA contributed to the increased oxidative stress burden in our model.

A recent study reported the phenotype of xanthine dehydrogenase (XDH)-stable and XO-locked knock-in mice^43^. Interestingly, the authors reported that XO knock-in mice showed significant increase in tumor growth compared with wild-type or XDH knock-in mice and that these effects were mediated by ROS production by XO knock-in macrophages. These data clearly indicate the physiological importance of XO/XDH balance in the immune system. Although we did not address XO or XDH activity in our study, previous evidence demonstrated that XO activity is elevated in the rat remnant kidney model^44^. The involvement of XO is also reported in other hypertensive kidney disease models^18,31,38^. Given these data, it is likely that the dysregulated XO activity and/or XO/XDH imbalance is involved in our model, which can be implicated in the cardio-renal protective effects of febuxostat observed in the study.

Another possibility that can potentially explain the protective effects of febuxostat is its effect on cellular ATP production. Indeed, several studies demonstrated that febuxostat alters ATP levels by activating purine salvage pathway^45,46^. In a recent study by Fujii *et al*.^45^, febuxostat promoted ATP recovery in the ischemia/reperfusion injury model, which was associated with the attenuation of kidney dysfunction. In another study, febuxostat increased intracellular ATP levels in bone marrow-derived macrophages, improving cellular bioenergetics^46^. Similar to the ischemia/reperfusion injury model, tissue ATP levels in the kidney is shown to be reduced in the remnant kidney rat^47^. Although we did not evaluate tissue ATP content in our study, the contribution of cellular bioenergetics in the protective effects in our model warrants further investigation.

In summary, our study demonstrated that the cardiac and renal injury in hyperuricemic CKD rats is attenuated by febuxostat. These data indicate the therapeutic potential of XOIs in cardiovascular and kidney dysfunction in hyperuricemic CKD, and also implicate XO in cardio-renal syndrome.

### Disclosures

Febuxostat was provided by Teijin Pharma. S.S. received research funding from Teijin Pharma. The company had no role in study design, execution of experiments, decision to publish, or preparation of the manuscript.

## Supplementary information

Supplementary information.
