# Supplementary figures and images for: Cardio-renal protective effect of the xanthine oxidase inhibitor febuxostat in the 5/6 nephrectomy model with hyperuricemia
Source: Sci Rep. 2020 Jun 9;10:9326. doi: 10.1038/s41598-020-65706-6 (PMC7283314; doi:10.1038/s41598-020-65706-6)

Supplemental Figure 1.  
Full-length images of Western blot analysis.

Fig. 6  
Nrf2

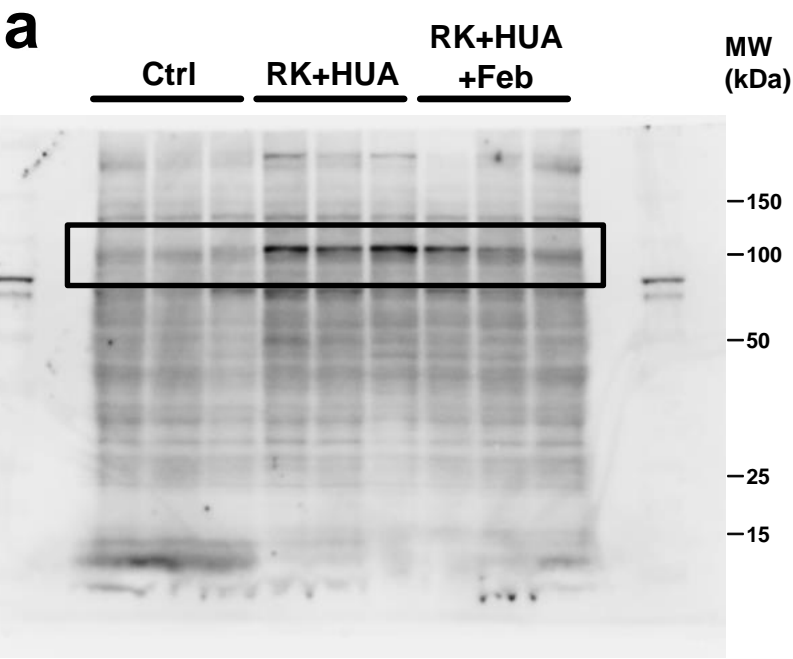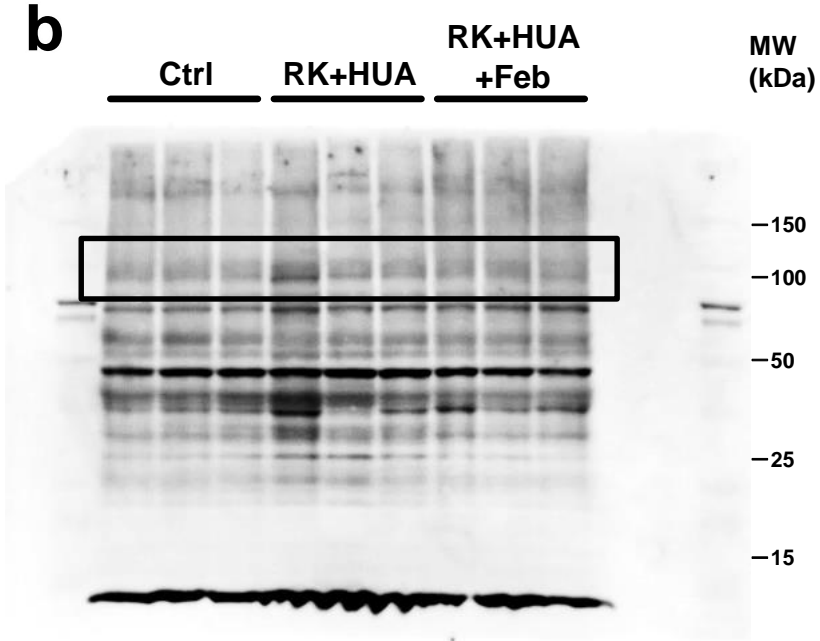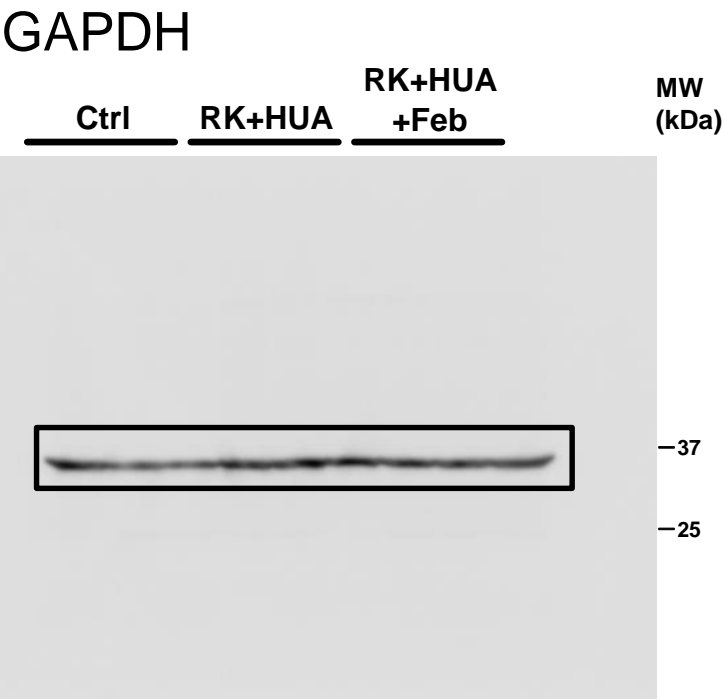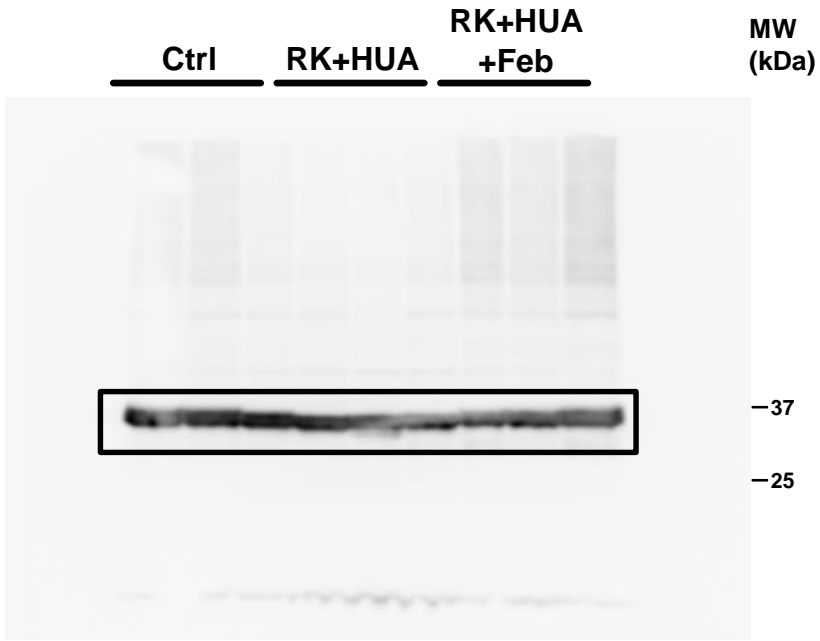

Fig. 6

HO-1

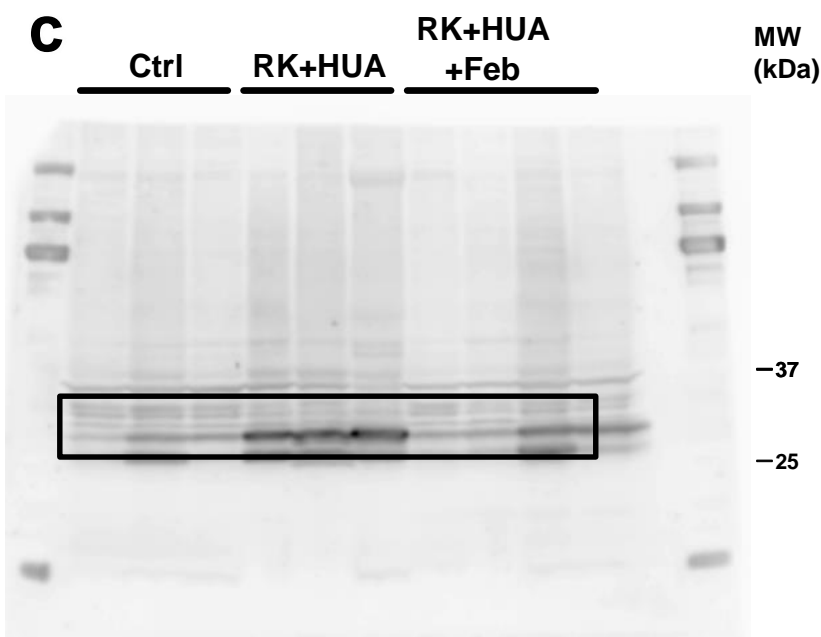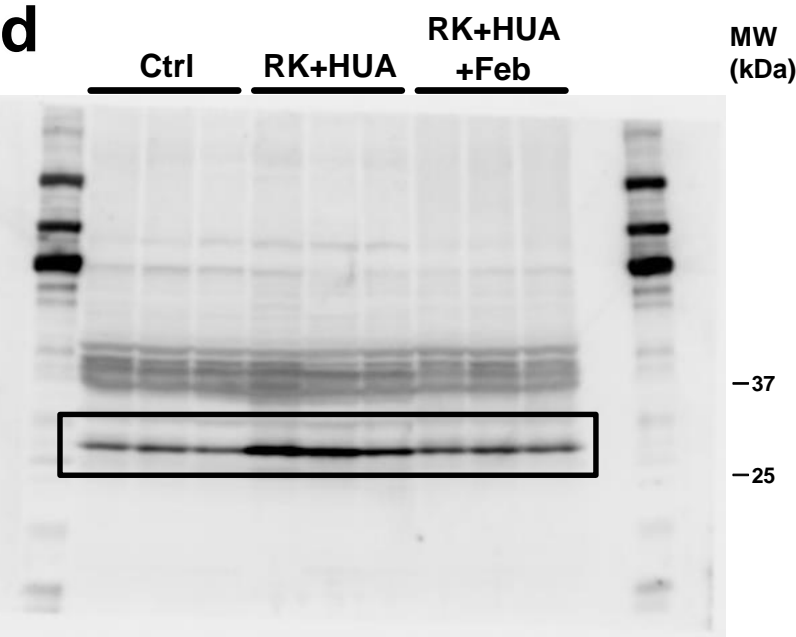

GAPDH

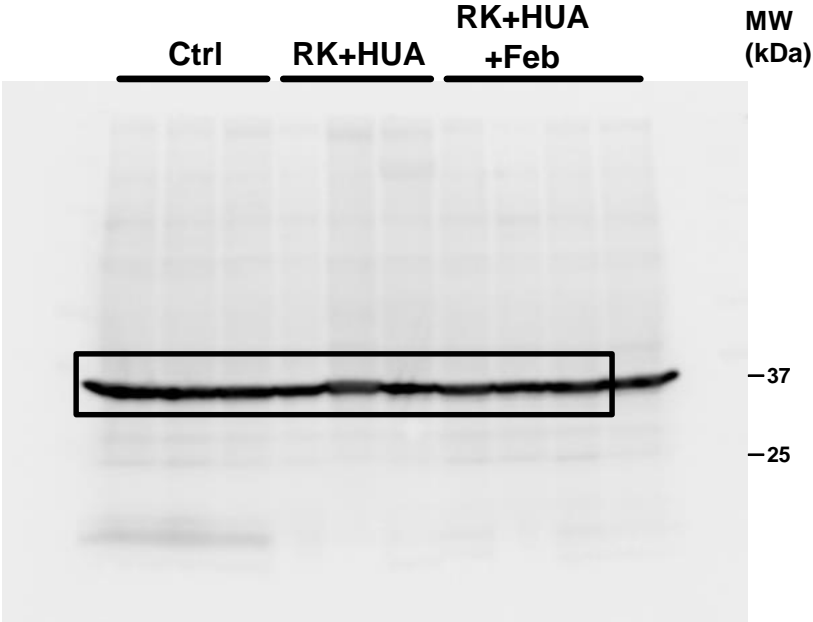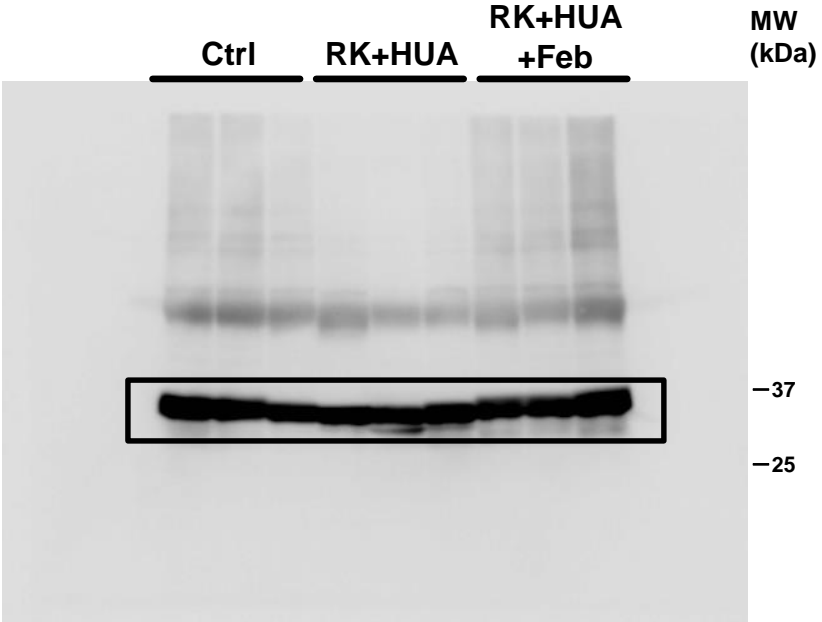

Supplement: Supplementary file 1 — Supplementary information. [file 41598_2020_65706_MOESM1_ESM.pdf]
